# Comparative Analysis of the Behavior of Llamas Housed in a Research Facility in Response to Different Environmental Enrichment Stimuli

**DOI:** 10.1111/asj.70201

**Published:** 2026-06-10

**Authors:** Raphael Brum, André de Abreu Rangel Aguirre, Klena Sarges Marruaz da Silva

**Affiliations:** ^1^ Plataforma de Criação e Experimentação Animal Fundação Oswaldo Cruz Porto Velho Brazil; ^2^ Instituto Oswaldo Cruz Fundação Oswaldo Cruz Rio de Janeiro Brazil; ^3^ INCT em Modelagem de Doenças Humanas Complexas com Plataformas 3D (INCT‐Model3D) São Paulo Brazil

## Abstract

Environmental enrichment (EE) is essential for promoting welfare and reducing atypical behaviors in captive research animals, but we know little about effective EE strategies for llamas ( *Lama glama* ). This study evaluated the behavioral responses to EE items provided to llamas housed in a biomedical research facility in Brazil, where they are maintained as biomodels for nanobody production. Eight EE items—including feeding, physical, and sensory stimuli—were tested through a longitudinal observational design supported by video analysis and ethograms. Descriptive statistics, repeated‐measures ANOVA, and cluster analysis (k‐means, hierarchical clustering, and principal component analysis [PCA]) were used to characterize behavioral patterns. Llamas showed a marked most time spent with the PVC pipe filled with hay or elephant grass, averaging 3.9 daily interactions. ANOVA indicated significant differences among animals (*p* < 0.001), with large effect sizes. Cluster analysis revealed two distinct behavioral profiles, confirming individual variation and identifying one llama with a unique interaction pattern. Sensory and recreational items (tire, grooming brush, and empty perforated ball) elicited low interaction, whereas forage‐containing items consistently ranked highest. These findings highlight the importance of forage‐based enrichment and demonstrate the value of behavioral analytics for designing dynamic EE programs that enhance welfare in llamas used in biomedical research.

## 1 Introduction

Llamas ( *Lama glama* ) are artiodactyls (Mammalia: Artiodactyla) belonging to the family Camelidae, naturally occurring in South America, predominantly at altitudes between 3800 and 5000 m above sea level, covering the central Andes region from central Peru to western Bolivia and northern Argentina. They are therefore also classified as South American camelids. These animals exhibit high adaptive capacity and can currently be found domesticated in several regions worldwide (Wilson and Mittermeier 2011; Wheeler 2012; Terry 2013). Their husbandry is one of the main agricultural activities of Andean communities, where they are valued for their meat, fur, manure, and use as pack animals (Szpak et al. 2014; Gandarillas et al. 2016). In Brazil, llamas can be found in parks and zoos, as well as in farms dedicated to fur production or cargo transport (Santos 2006).

In addition to their use in livestock production, camelids (camels, dromedaries, llamas, and alpacas) are also used in biomedical research because they produce IgG antibodies devoid of light chains. These antibodies contain only antigen‐binding domains derived from heavy‐chain immunoglobulins, known as single‐chain antibodies. These antibodies are attractive for antibody engineering, resulting in the production of only the variable region (Fab), referred to as VHHs or nanobodies. Owing to their small size, nanobodies can recognize epitopes (T‐cell receptors that enable identification and defense against infectious microorganisms such as bacteria and viruses) inaccessible to conventional antibodies and also exhibit high stability under extreme conditions, high solubility, ease of cloning, and low production cost (Minatel et al. 2024). For this reason, since the discovery of single‐chain antibodies in these animals and the subsequent development of camelid nanobodies, the breeding of llamas and other camelids has expanded into biomedical research facilities (Hamers‐Casterman et al. 1993), and these animals have been widely used in research areas related to diagnostics and pharmacotherapy. The unique properties of nanobodies, such as small size, high stability, strong antigen‐binding affinity, water solubility, and natural origin, make them suitable for the development of biopharmaceuticals and nanoreagents (Pedreáñez et al. 2021).

As with any animal used in research, llamas require environmental enrichment (EE) in their housing conditions to promote improved welfare and quality of life, stimulating their activities and reducing atypical or stereotypic behaviors that would not typically occur in their natural environments (Damasceno 2018).

The use of EE for llamas on a farm belonging to a Brazilian university was reported by Quintão and Repetti (2023). As feeding enrichment, they provided rocket (arugula) and chicory hung on a clothesline placed between trees in the enclosure, in addition to tires used as feeders suspended by ropes. As physical enrichment, they used brooms made of straw for self‐grooming. The study involved only three animals but reported that llamas behaved more calmly, allowing contact, interaction, and human approach after the period during which enrichment items were provided.

However, there is limited available literature on the behaviors of llamas and the appropriate items for implementing an EE program. Most publications originate from Andean countries and are generally limited to academic or technical reports (de la Miranda‐ Lama et al. 2022). The most comprehensive available review on the behavior and welfare of llamas raised for agricultural purposes was conducted by Miranda‐de la Lama and Villarroel (2023). For llamas maintained in research facilities, reference material or studies describing their behavior under such conditions is even more scarce.

The Oswaldo Cruz Foundation (Fiocruz), a Brazilian public health research institution, has one of its units located in Porto Velho (Rondônia, Brazil), in the northern region of the country, known as Fiocruz Rondônia. This unit maintains a herd of llamas used for studies focused on the development of camelid‐derived nanobodies. Motivated by the importance of this biomodel for public health research, this study was conducted to support the development of environmental enrichment methods that promote the welfare of these animals housed in research animal facilities.

## 2 Materials e Methods

Five adult llamas—four males aged between 10 and 15 years (Animals 1, 2, and 3) and one aged 4 years (Animal 4) and one female (13 years old—Animal 5) from the herd of the Animal Breeding and Experimentation Platform (PCEA) at Fiocruz Rondônia were used in this study. Three animals were acquired from a breeding facility located in Saudade do Iguaçu (Paraná, Brazil), one from the city of Gravataí (Rio Grande do Sul, Brazil), and one was born at PCEA/Fiocruz Rondônia.

The animals were housed in four 12 × 12 m corrals (A‐B‐C‐D), partially covered with roofing tiles, equipped with feeders and drinkers made from polyethylene barrels. During the study period, Animals 1 and 2 were housed together in Corral C, Animal 3 in Corral D, Animal 4 in Corral B, and Animal 5 in Corral A. This animal allocation configuration was maintained prior to the study and was kept in place to avoid interpretation biases.

Llamas were fed daily with Tifton‐85 hay (*Cynodon* sp.) and elephant grass ( *Pennisetum purpureum* ), in addition to equine feed containing 11% crude protein and 18% fiber, supplied at 0.5%–1% of body weight per day. Water was provided ad libitum.

### 2.1 Environmental Enrichment Items

Eight different environmental enrichment (EE) items were used, comprising feeding and sensory enrichment: Hay made from grasses and legumes, elephant grass, mineral salt for equines, common quicklime, 50‐cm‐diameter spheres with 2 cm openings produced via 3D printing using PLA filament (a nontoxic and biodegradable material), a recycled 200‐mm white PVC pipe, repurposed size‐14 rubber tires, netted bags (90 cm long; 3 mm rope), and a stiff‐bristle grooming brush.

The perforated balls, PVC pipes, tire, and net were introduced into the pens individually and each remained for 2 months, alongside the grooming brush, forage, mineral salt, and quicklime, which were continuously available because they were already used in routine husbandry prior to the study. The tire and grooming brush were tied to support pillars in the sheltered area of the pens to encourage body rubbing. The PVC pipe was positioned next to the feeder, and the net was hung 1 m above the ground from the roofing structure; both were filled daily with elephant grass and hay, respectively. The spheres were suspended 1.5 m above the ground. Each pen received two spheres: one empty and one filled with forage (elephant grass).

The order in which the items were introduced was randomized to alternate the order in which the items were offered to each animal.

Mineral salt, used as a dietary supplement, and quicklime, used as a sanitizing agent, were spread on the pen floor before the feeding period.

### 2.2 Behavioral Assessment and Data Analysis

A longitudinal observational study was conducted to evaluate the animals' interaction with the enrichment items using an ethogram adapted from Albuquerque and Codenotti (2006). The ethogram was completed through video analysis from two cameras installed near the pens. Observations occurred between 5:00 and 7:00 a.m. on alternating days. The recording sessions with the cameras were made simultaneously for all corrals and animals. Interactions with items containing or not containing forage were recorded based on 2 h of daily filming, obtained three times a week, totaling approximately 360 h of recordings over 3 months of study, resulting in the same cumulative time of observation for each animal. Interaction was defined here as any action indicating that the animal was interested in the environmental enrichment object, such as voluntarily approaching to examine the object, smelling, touching, feeding on the forage contained in it, and playing. Involuntary contact with the object in the enclosure, such as bumping into it, was not included as interaction.

The data obtained from the ethogram and tabulated in Excel were analyzed using descriptive statistics. Normality was assessed using the Shapiro–Wilk test. To identify significant differences in interaction frequency among the items, a repeated‐measures Analysis of Variance (ANOVA) was applied, considering only the interactions involving the perforated ball, PVC pipe, tire, and net.

Additionally, cluster analysis was performed to assess behavioral patterns associated with the items evaluated in the ANOVA, as well as other items not included in the initial statistical analysis (grooming brush, mineral salt, and quicklime), to compare possible interactions not observed and analyzed prior to this study with items already known to the animals. k‐means and hierarchical (Ward method) clustering were used. Cluster validation and optimal cluster number were determined using internal validation metrics (Silhouette Score) and principal component analysis (PCA). In the PCA, PC1 represented the contrast between the non‐ANOVA items (quicklime, grooming brush, and mineral salt) and the ANOVA items, whereas PC2 captured differences among the perforated ball, PVC pipe, and net. Identification of two distinct behavioral clusters (PC1 and PC2) demonstrated variation in individual behavioral profiles and revealed a unique behavioral pattern in one animal. Cluster analyses were conducted using the machine learning libraries and data analysis Python (v3.11) and the Scikit‐learn, SciPy, and Panda's libraries.

## 3 Results

An observed behavior, not listed on the ethogram, confirmed a behavior previously reported by the staff responsible for animal care. Immediately after the installation of enrichment items, the llamas remained standing still or lying down for some time before approaching the newly provided items. This behavior had already been observed during routine husbandry whenever new food or objects were introduced.

**FIGURE 1:**
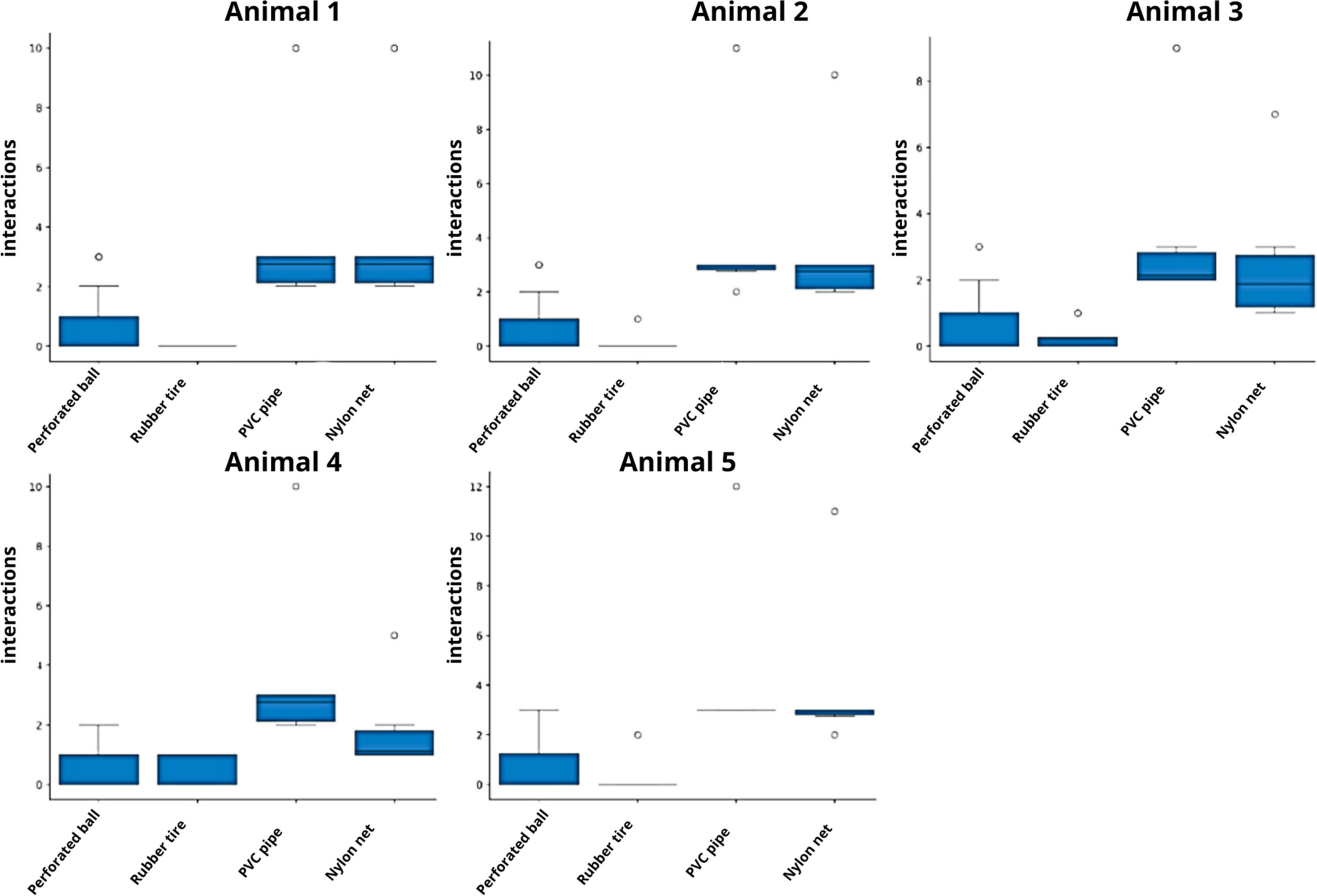
Individual ANOVA results comparing interactions with the items punctured ball, pipe, net, and tire between llamas kept at PCEA/Fiocruz Rondônia (Brazil).

Descriptive analysis of ethogram data referring to interactions with the perforated ball, PVC pipe, tire, and net showed that all items were used (Figure 1). However, the llamas exhibited more time interacting with the PVC pipe containing hay/forage, with a mean of 3.9 daily interactions (Table 1).

**TABLE 1:** Average individual and overall interaction of animals in relation to the items pipe, net, punctured ball, and tire.

Animal	PVC pipe	Netted bags	Perforated balls	Tire
**1**	4.12 (±3.39)	3.75 (±3.09)	0.62 (±1.03)	0.12 (±0.35)
**2**	3.75 (±3.09)	1.87 (±1.57)	0.55 (±0.57)	0.37 (±0.51)
**3**	4.50 (±3.67)	4.12 (±3.39)	0.80 (±1.01)	0.25 (±0.70)
**4**	3.37 (±2.78)	2.62 (±2.26)	0.64 (±0.88)	0.25 (±0.46)
**5**	3.75 (±3.09)	3.75 (±3.09)	0.62 (±0.88)	0.00
*Average of interactions*	**3.90**	**3.22**	**0.64**	**0.20**

*Source:* Author's own work, 2025.

**FIGURE 2:**
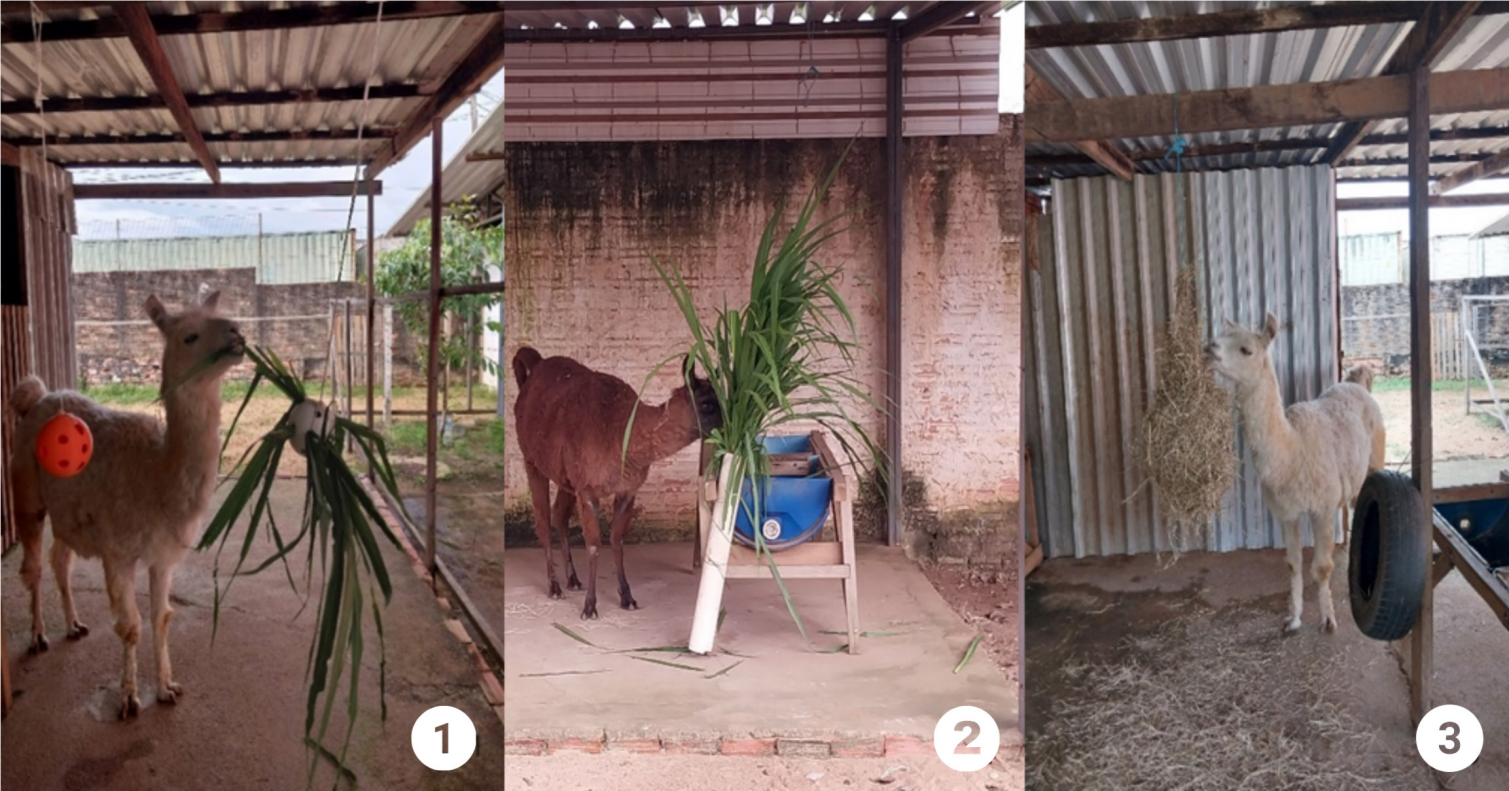
Llamas kept at PCEA/Fiocruz Rondônia (Brazil) using the environmental enrichment items evaluated in the study (1‐ perforated ball; 2‐ PVC pipe; 3‐ tire, and net).

The ANOVA revealed highly significant individual differences (*p* < 0.001) among the items explored by each animal, with partial eta squared (*η* ^2^_*p*_) values ranging from 0.360 to 0.442, indicating large effect sizes (*η* ^2^_*p*_ > 0.14). The longest interaction time observed with the PVC pipe with hay/forage was confirmed for all animals (Figure 2).

All groups showed significant deviations from normality (*p* < 0.05 in the Shapiro–Wilk test), a result commonly expected in behavioral datasets. Nonetheless, ANOVA is considered robust to nonnormality violations.

### 3.1 Cluster Analysis Results

Cluster analysis identified groupings that supported more time in interactions with the PVC pipe containing hay/forage, suggesting the presence of individual behavioral patterns in llamas. In the validation metric based on the dissimilarity matrix, the original matrix with *k* = 2 displayed the best Silhouette Score (0.570), indicating well‐defined and adequately separated clusters.

In the PCA, PC1 accounted for 52.4% of the total variance and PC2 accounted for 34.4%. Combined, PC1 + PC2 explained 86.8% of the total variance, representing an excellent two‐dimensional representation.

**FIGURE 3:**
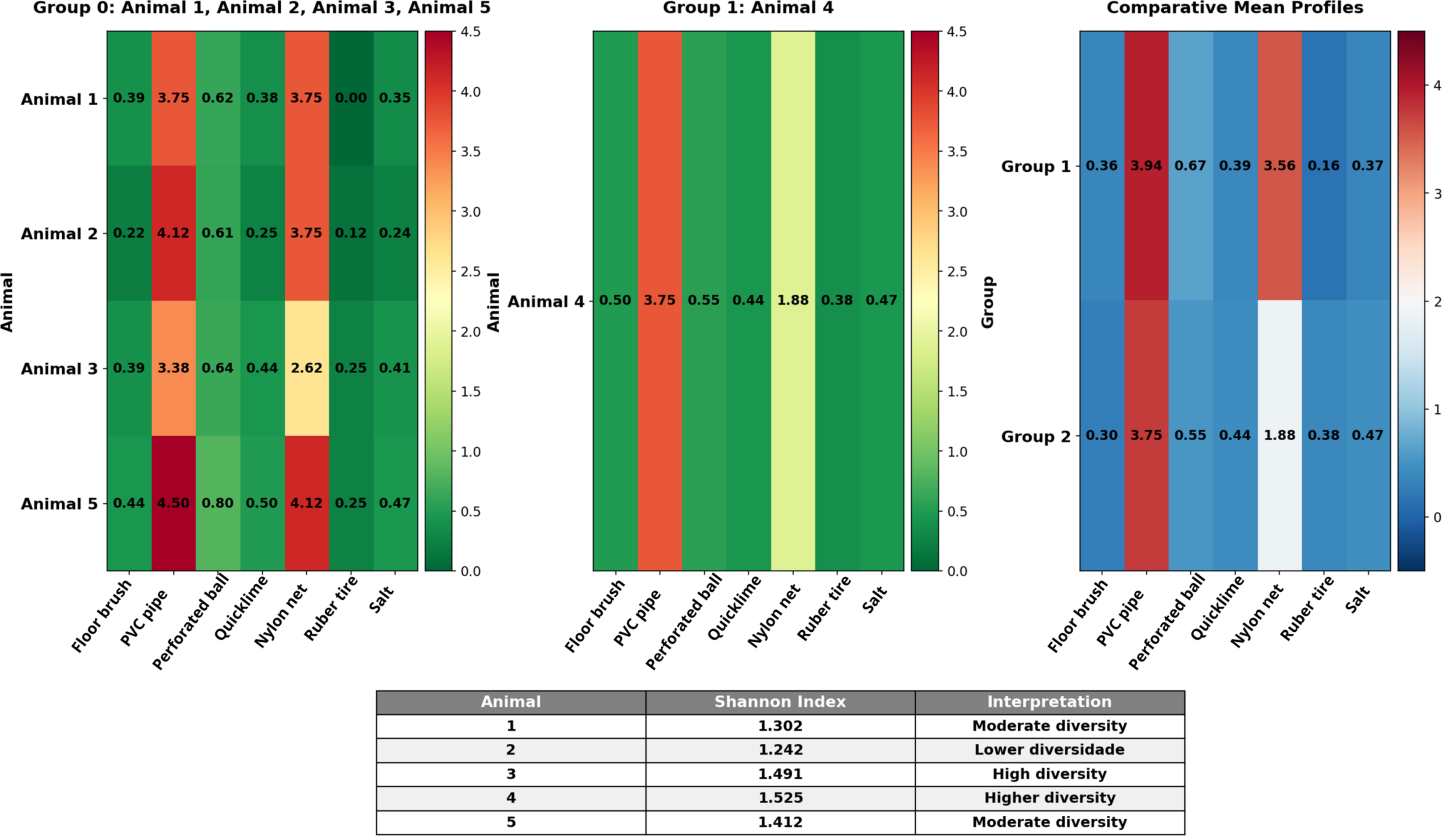
Interaction Average Matrices by Animal and Item. The first matrix represents the interaction pattern with the items observed for animals 1‐2‐3‐5 (Group 0). The second matrix represents the differentiated behavioral pattern of Animal 4 (Group 1) in the interaction with the items. The third matrix represents the comparative average profiles between them.

In matrix‐based validation, the configuration with *k* = 2 yielded the best Silhouette Score (0.570), indicating clear cluster separation. Regarding the Intensive Structural Profile (Optimal Configuration: *K* = 2), low interaction with the other items (mineral salt, quicklime, and grooming brush) was observed (mean = 0.37) for Animals 1, 2, 3, and 5, with moderate within‐group variability (0.23). The most time spent for the seven items assessed was PIPE > NET > PERFORATED BALL > QUICKLIME > MINERAL SALT > GROOMING BRUSH > TIRE, confirming results from the descriptive analysis and ANOVA. All animals, regardless of individual interaction patterns, showed the most time spent for the PVC pipe containing hay/forage (Figure 3a). Animal 4 exhibited a different behavioral pattern compared to the others (Shannon Index = 1.525), with lower time spent for the net (mean = 1.88 vs. the group mean of 3.56) (Figure 3b).

Additionally, interaction with the pen substrate was observed throughout the study period, either by rolling on the ground itself or specifically on the quicklime and mineral salt provided as enrichment items.

## 4 Discussion

The consistent hierarchical pattern in llamas' use of environmental enrichment items—showing a longer interaction time for interacting with the PVC pipe, followed by the net, perforated ball, and lastly the tire—may be strongly related to the inclusion of elephant grass as forage inside the PVC pipe. This forage was offered fresh immediately after collection, in addition to hay, which is a volumetric feed and a key dietary component for these camelids. Erdoğan et al. (2016) observed that llamas preferentially select diets composed predominantly of tall grasses that form dense tussocks, and they also feed on available shrubs and trees. Thus, the use of freshly harvested tall grasses inserted into enrichment items is a strong candidate for “golden items” enrichment in llamas. The PVC pipe containing elephant grass simulates a natural tussock, which may have contributed to observing a greater interest. It is important that future studies explore additional combinations regarding different presentation formats of forage types (fresh grass vs. dried hay), PVC pipes of different heights, spheres and nets of varying sizes, and installation at different heights to determine more precisely the most effective way to offer volumetric feed to llamas in captivity.

Interaction with the perforated ball when offered with food has also been reported in horses by Bitti (2020). Horses using perforated balls in EE studies displayed distraction behaviors that helped prevent the development of stereotypies, repetitive behaviors, and continuous feeding. However, the perforated ball without food—offered to llamas to stimulate exploratory behaviors such as pushing, butting, or other forms of play—showed low use, with no clear recreational interactions. It was noted that during the first days after the balls were introduced into the pens, the llamas were hesitant to approach them, likely because the objects were unfamiliar. Over time, the animals approached the empty balls but did not interact with them. The few recorded occurrences (*n* = 15) were incidental, as the item was placed near the water trough.

Both alpacas and llamas can display fearful and aggressive behavior in farm settings when near humans. Observed behaviors include spitting, biting, vocalizing (screaming/howling and snorting/clicking), kicking, abruptly lying down on the ground, and behavioral freezing (Windschnurer et al. 2020). It is possible that the hesitation to interact with novel items, as seen with the perforated ball, reflects the natural behavior of the species when faced with unfamiliar people or objects. Additionally, vocalization and kicking behavior were observed in the male llamas of this study prior to the introduction of forage‐containing items.

Although the study included items intended for recreational (perforated ball without food) and sensory (tire and grooming brush) enrichment, it is recommended that interactions with these items be evaluated separately from assessments of feeding enrichment, as llamas showed a clear interest for items containing forage. Studies specifically focused on recreational and sensory enrichment could help clarify llamas' interest in such items in captivity, improving understanding of behaviors related to rolling on substrates and even allowing the design of new objects produced via 3D printing, customized based on observed positive‐stimulus behaviors. The tendency to scratch against hard surfaces such as posts, beams, and walls, as well as to wallow in dusty areas (dust baths), has been documented in other camelids (Matthews et al. 2020). South African camelids exhibit scratching and rolling behavior to maintain the insulating properties of their fur and remove parasites (Aba et al. 2010). However, communal use of these substrates is also a potential source of external parasite transmission within herds (Van Hoy et al. 2022).

Beyond the identification of greatest interest items, it is equally important to investigate the stability of interactions over time to support the development of a dynamic EE program, with adequate rotation frequency of items to ensure sustained exploratory activity and animal welfare throughout their lifespan in research facilities. It is important to highlight that multivariate analyses should be interpreted as exploratory within the studied population and that further studies with larger cohorts are warranted to confirm generalizability. In such studies, the use of cluster analysis techniques—such as those applied here—is recommended. Despite the small number of animals, the analysis demonstrated that it is possible to identify distinct behavioral patterns among individuals within the same group of llamas housed in research settings.

## Conflicts of Interest

The authors declare no conflicts of interest.

## Data Availability

The data that support the findings of this study are available from the corresponding author upon reasonable request.
